# Evaluation of intestinal leakage pressure following unsutured intestinal biopsies performed with an automatic needle-core biopsy device

**DOI:** 10.3389/fvets.2026.1870783

**Published:** 2026-07-20

**Authors:** Esther Deglume, Roberta Aloisi, Guilhem Delsol, Rachel Malkani, Stéphane Libermann, Sébastien Etchepareborde

**Affiliations:** 1 IVC Evidencia, CHV des Cordeliers, Meaux, France; 2 Clinique Vétérinaire AniCura VetRef, Beaucouzé, France; 3 Department of Research, IVC Evidensia UK, Bristol, United Kingdom

**Keywords:** biopsy, full-thickness biopsy, intestine (small), leakage pressure, needle-core biopsy

## Abstract

**Objective:**

To evaluate whether full-thickness intestinal biopsies performed with an automated needle-core device, without closure of the biopsy site, maintain adequate intestinal integrity in dogs and cats.

**Materials and methods:**

This experimental study consisted of *ex vivo* testing on small intestinal segments from fresh canine and feline cadavers, and *in vivo* testing in dogs and cats undergoing enterectomy for unrelated indications. Full-thickness biopsies were performed using a 16-gauge automated needle-core device, with biopsy sites left unsutured. Intraluminal pressure was progressively increased using methylene blue–stained saline, and the initial leakage pressure (ILP) was recorded at first visible evidence of leakage. Cadaveric and *in vivo* ILP were compared to each other and to the reported maximal physiological intraluminal pressure of 25 mmHg.

**Results:**

Thirty-nine cadaveric intestinal segments from seven animals were evaluated. The mean cadaveric ILP was 20.7 mmHg (95% CI [5.9–35.5 mmHg]), with leakage observed below 25 mmHg in 33 of them. *In vivo* ILP was measured in 12 patients (8 dogs, 4 cats), yielding 23 measurements. The mean *in vivo* ILP was 85.66 mmHg (95% CI [55.53, 115.78]), significantly exceeding the reference value of 25 mmHg, and all measurements remained above 26 mmHg.

**Conclusion:**

Cadaveric leakage pressures were markedly lower than *in vivo* measurements, suggesting that cadaveric models may be poorly suited for evaluating intestinal pressure. In contrast, unsutured needle-core biopsy sites in live dogs and cats tolerated pressures above reported maximal physiological levels, immediately after biopsy. These findings suggest that the immediate risk of leakage at small (1.3-mm) unsutured biopsy sites is low; however, because healing and longer-term integrity were not evaluated, the necessity of routine closure cannot be established from these data. Further studies are needed to evaluate long-term safety and applicability in diseased intestines.

## 1 Introduction

Intestinal biopsies are frequently performed in veterinary medicine for multiple diagnostic purposes (1–7). For reliable histological evaluation, full-thickness biopsies are often required, allowing distinction between various chronic intestinal diseases such as inflammatory bowel disease (lymphoplasmacytic, eosinophilic, or neutrophilic), lymphangiectasia, and intestinal lymphoma (1–7). Partial-thickness biopsies obtained via endoscopy offer lower diagnostic accuracy compared with full-thickness biopsies (1–3). In addition, alimentary lymphoma in cats mainly affects the jejunum and ileum, areas that are more difficult to sample endoscopically (3).

Despite their diagnostic advantages, full-thickness intestinal biopsies are associated with potential complications, the most serious one being dehiscence of the biopsy site leading to septic peritonitis. Reported rates of postoperative dehiscence following full-thickness biopsy range up to 11%, although the risk appears lower in cats than in dogs (5–8). Consequently, the decision to perform full-thickness biopsy requires careful consideration of diagnostic benefit versus surgical risk.

Recently, veterinary medicine has been moving toward developing minimally invasive techniques. Full-thickness intestinal biopsies are traditionally obtained via laparotomy or laparoscopic-assisted approaches (9–11). Although laparoscopic-assisted techniques reduce incision size and postoperative pain, studies comparing these approaches with laparotomy have not consistently demonstrated differences in perioperative complication rates (9–12). Traditionally, intestinal biopsies have been performed using an incisional biopsy or a punch biopsy (5, 6, 13). However, needle-core biopsy via laparotomy has recently been evaluated in dogs and cats (7). This technique offers several advantages, including reduced surgical time, low complication rates, and a good histological quality (7). In the reported series, no postoperative dehiscence occurred; however, all biopsy sites were routinely sutured (7).

Given the small diameter of needle-core biopsy sites (approximately 1.3 mm), the necessity of routine closure remains uncertain. Eliminating suturing could further reduce invasiveness and potentially facilitate alternative sampling techniques, such as laparoscopic, percutaneous, or ultrasound-guided intestinal biopsies. However, the safety of leaving needle-core biopsy sites unsutured depends on whether adequate intestinal integrity is maintained under physiological intraluminal pressures.

Several studies have documented intraluminal intestinal pressure in dogs and cats (14, 15). The small intestine is a low-pressure system, with resting physiological pressure values typically ranging from 2 to 4 mmHg (14, 15). During peristaltic activity, pressures can rise to between 15 and 25 mmHg (14–17). These values vary depending on the stage of digestion and on whether the animal is under anesthesia. An earlier study measuring intestinal pressure in unanesthetized dogs reported an average physiological pressure of about 6 mmHg throughout the small intestine (18).

The objective of this study was to compare the initial leakage pressure (ILP) following unsutured intestinal biopsy with reported physiological intraluminal pressures in dogs and cats, as an indicator of the immediate pressure resistance of the biopsy site. This pressure was evaluated on the intestines of fresh cadavers, as well as on intestines of alive animals. We first hypothesized that the intestinal pressure measurements obtained would be comparable between a cadaveric and *in vivo* models. We then hypothesized that the initial leakage pressure following a digestive biopsy performed with an automated device, without site closure, would exceed physiological intestinal intraluminal pressures in dogs and cats.

## 2 Materials and methods

This study was approved by the Jacques Bonnod Ethics Committee of VetAgro Sup (No. 2503).

### 2.1 Ex vivo

Jejunal segments were collected from adult dogs and cats that had died or were euthanized for reasons unrelated to the study. Animals were excluded if there had been any known history of gastrointestinal disease. In all cadavers, the entire gastrointestinal tract was systematically examined, and specimens were excluded if any macroscopic abnormalities were identified. Pressure testing was performed on segments collected from all three parts of the small intestine (duodenum, jejunum, and ileum), as these are the most commonly sampled sites during full-thickness intestinal biopsy in dogs and cats.

Multiple intestinal segments of 10 cm in length were harvested from fresh cadavers within 6 h of death. Each intestinal segment was thoroughly rinsed and emptied of luminal contents. Both ends of each segment were occluded using Doyen forceps. Segments were tested immediately after harvesting, with no intervening refrigeration or storage.

Full-thickness biopsies were obtained as previously described by Maggiar et al.: an automated 16-gauge needle-core biopsy device was inserted into the intestinal lumen at a 45° angle relative to the antimesenteric border. After each sampling, biopsy specimens were grossly evaluated as qualitative but were not preserved for histological analysis. Biopsy sites were left unsutured.

Two 18-gauge IV catheters were then inserted obliquely into the intestinal lumen. The methylene blue–stained saline was infused through one catheter at a continuous flow rate of 500 mL/h, while intraluminal pressure was continuously measured via a pressure transducer connected to the second catheter. The pressure at which initial leakage was visually detected was recorded and defined as the ILP, expressed in mmHg. A single pressure measurement was obtained for each intestinal segment tested. The infusion was stopped as soon as the first leak occurred.

### 2.2 In vivo

The study was conducted in dogs and cats undergoing enterectomy at our institution between February and October 2025. All surgical procedures were performed for clinical indications unrelated to the study, including obstructive intestinal masses, gastrointestinal foreign bodies, and intestinal perforations. Only intestinal segments scheduled for surgical resection were tested. All procedures were performed by the same surgical resident under the supervision of a board-certified surgeon.

**Figure 1:**
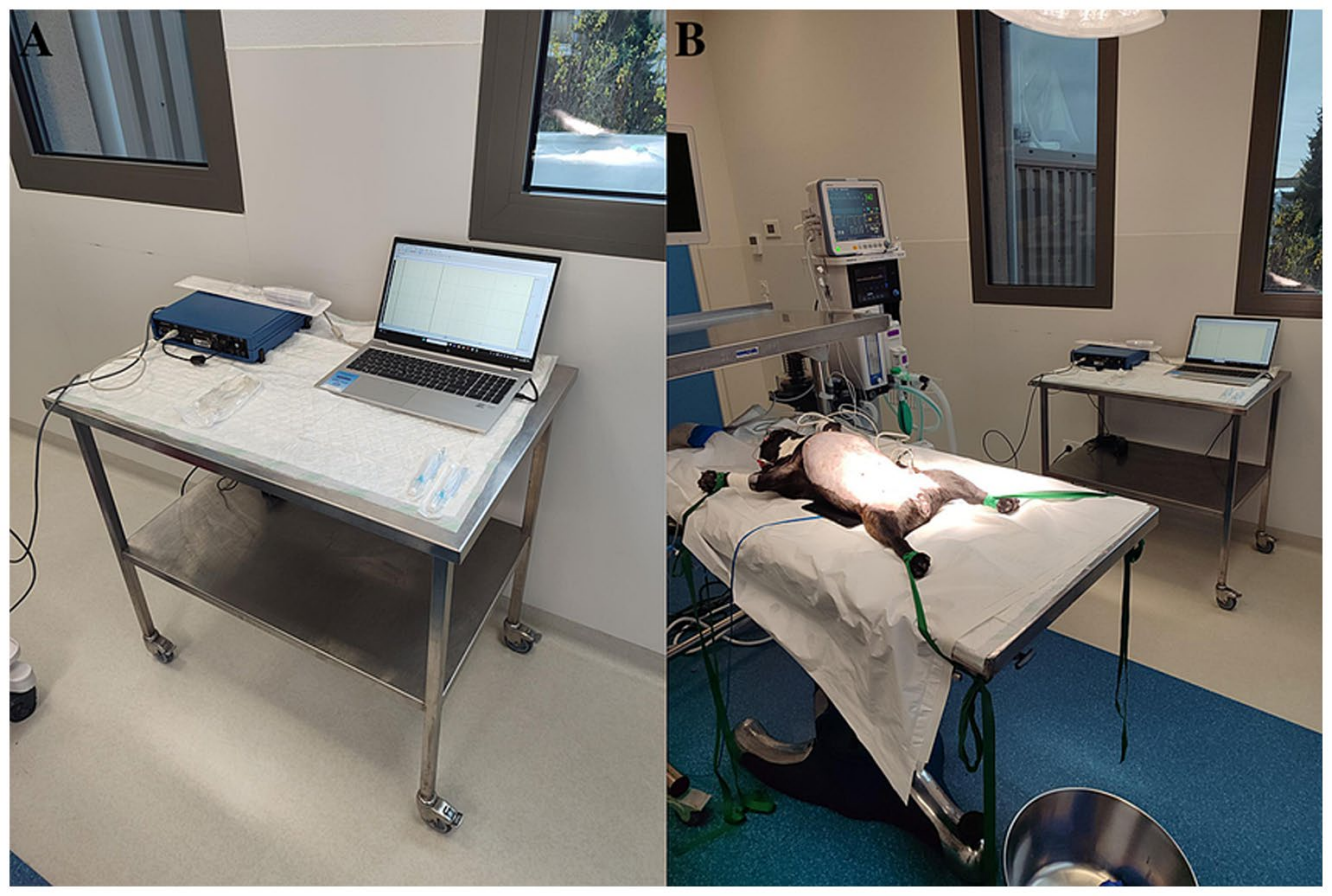
Experimental setup for intraluminal pressure measurement **(A)** and operating room organization during the procedure **(B)**.

The animals were anesthetized using protocols selected at the clinician’s discretion and adapted to each patient’s clinical condition. Following induction, they were positioned in dorsal recumbency, clipped, and aseptically prepared for a standard ventral midline celiotomy. To minimize additional anesthetic time, all experimental equipment and connections were prepared in advance in the operating room prior to positioning the animal on the surgical table (Figure 1).

A ventral midline laparotomy was performed, and the entire peritoneal cavity was systematically explored. The intestinal lesion was identified and isolated using a surgical drape. The experimental procedure was conducted on vascularized intestine before enterectomy. Full-thickness biopsies were obtained from macroscopically normal intestinal tissue located both oral and aboral to the lesion requiring resection. An intestinal segment was isolated upstream and downstream of the lesion using two Doyen forceps, thereby delimiting a segment of visually healthy tissue. The biopsy was performed at the center of this segment, at a distance from both forceps.

**Figure 2:**
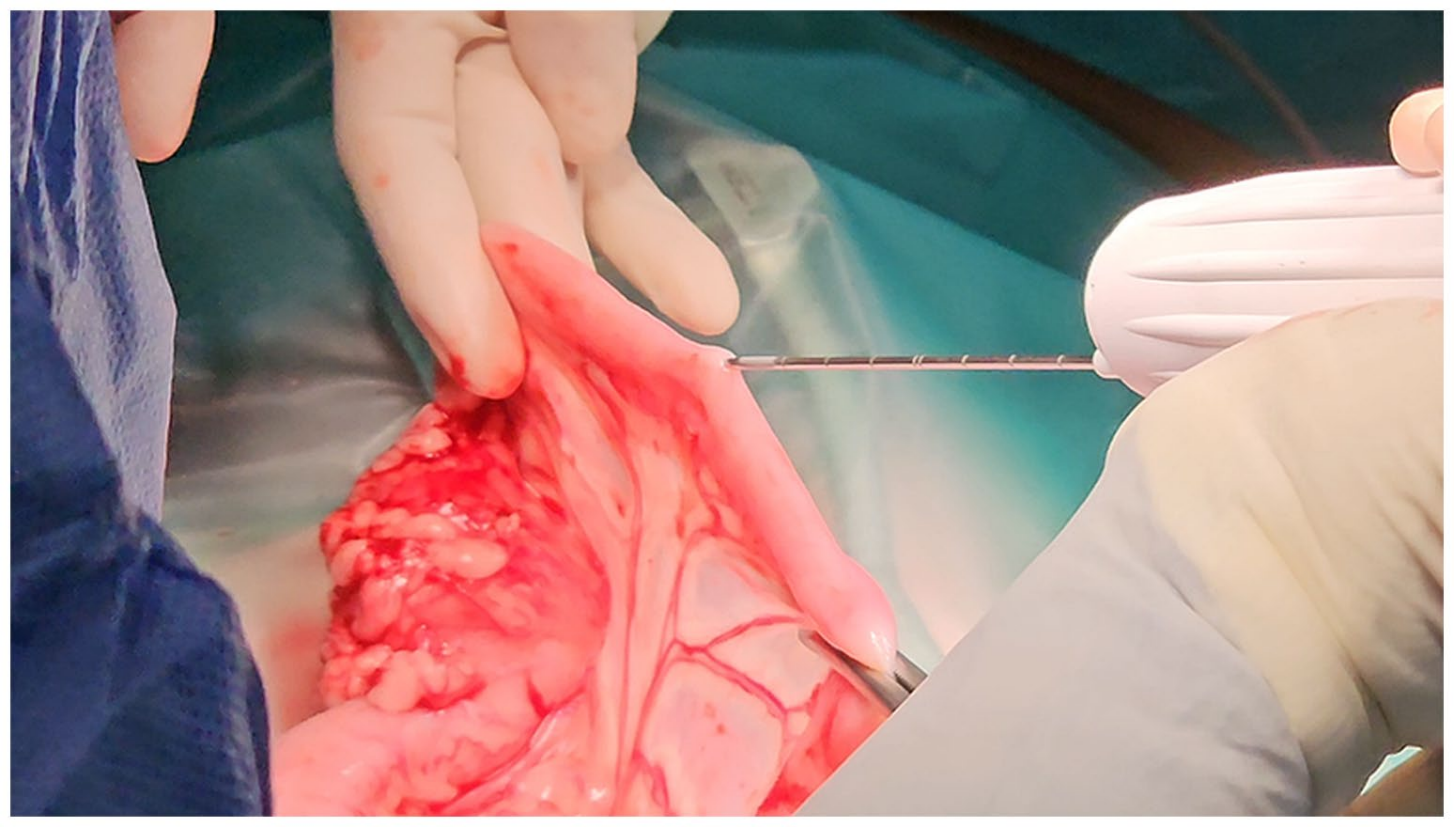
Full-thickness intestinal biopsy performed by using an automated 16-gauge needle-core biopsy device inserted into the intestinal lumen at a 45° angle relative to the antimesenteric border.

As in the *ex vivo* ones, the biopsies were performed as previously described by Maggiar et al. (7) using a 16G automated needle-core biopsy device inserted into the intestinal lumen at a 45° angle relative to the antimesenteric border (Figure 2). Biopsy specimens were visually assessed for qualitative adequacy. The 1.3-mm biopsy sites were left unsutured and isolated using two Doyen intestinal forceps.

**Figure 3:**
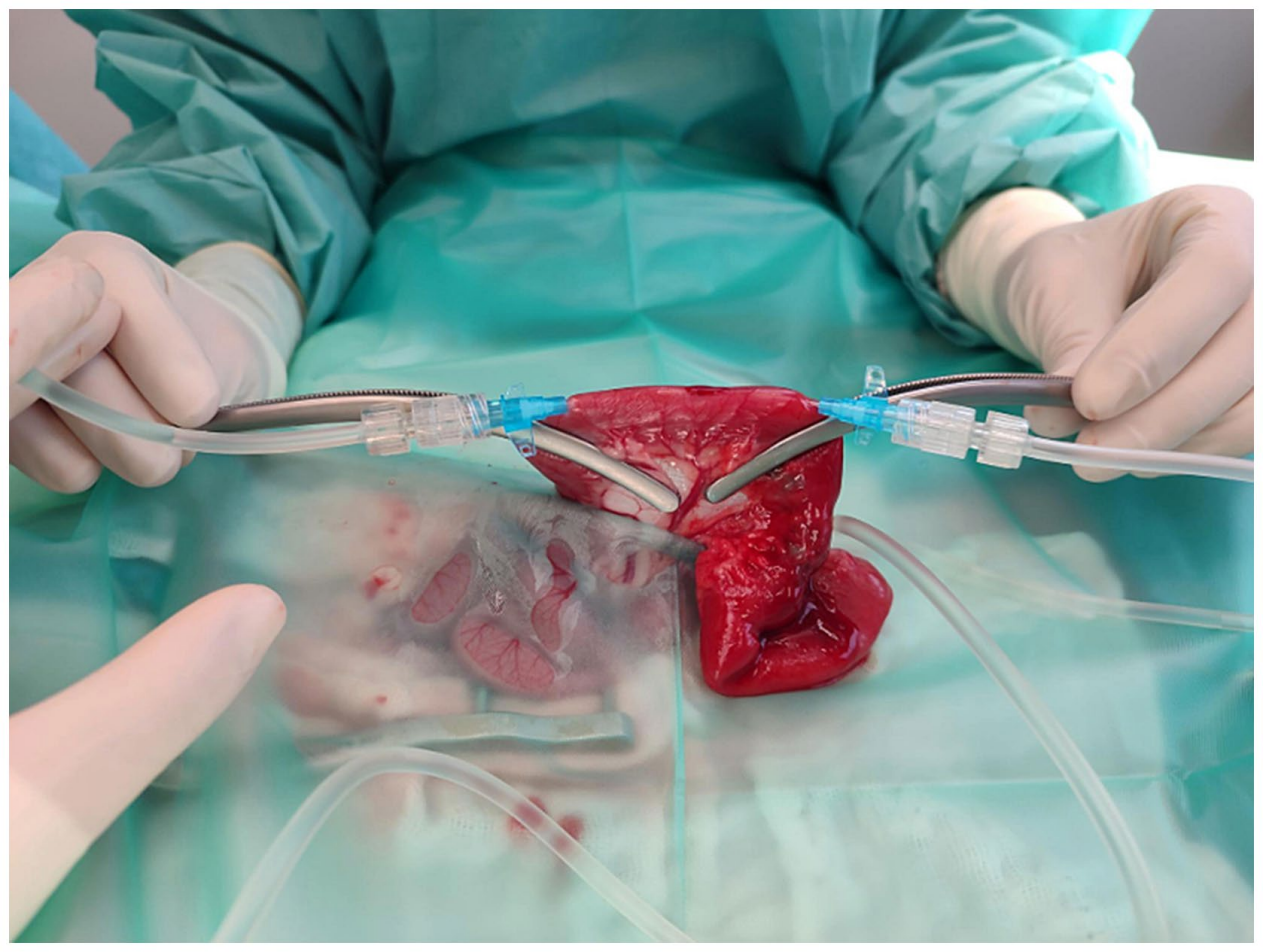
The sampling site was left unsutured and isolated with 2 Doyen clamps. Two 18-gauge IV catheters were inserted into the intestinal lumen: a methylene blue–stained saline solution was infused through the left catheter, while the intraluminal pressure was recorded using a transducer connected to the right catheter.

Two 18-gauge IV catheters were inserted obliquely into the intestinal lumen. The methylene blue–stained saline solution was infused through one catheter at a constant rate of 500 mL/h, while intraluminal pressure was continuously recorded via a pressure transducer connected to the second catheter (Figure 3).

**Figure 4:**
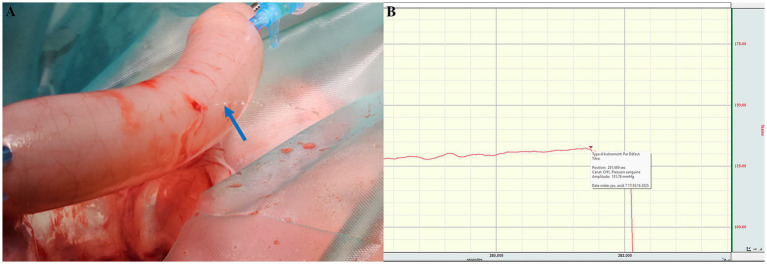
The intestine was progressively inflated until leakage was observed (blue arrow) **(A)**. The initial leakage pressure corresponded to the maximal pressure recorded on the monitor either at the appearance of a plateau or at a subsequent decrease in pressure **(B)**.

The ILP was recorded for each test and corresponded to the first macroscopically visible leak. Methylene blue was used to facilitate early detection of leakage. This ILP was typically associated with a plateau or a decrease in pressure recorded by the pressure transducer (Figure 4). Afterwards, the infused fluid was aspirated, and the enterectomy was performed, removing the primary lesion as well as both biopsy sites.

Recorded ILP values were compared with previously reported physiological intestinal intraluminal pressures and data obtained in the cadaveric study.

Although viable intestinal tissue was required for testing, all intestinal segments used in the experiment were scheduled for surgical removal. The experimental procedure required only a few minutes to complete and did not result in any residual effects within the abdominal cavity. Consequently, postoperative care and monitoring of the animals remained identical to those routinely provided following enterectomy thus were not affected by participation in the study. The study did not alter postoperative pain or increase the risk of postoperative complications. For this reason, postoperative follow-up, clinical outcomes, and postoperative complications were not evaluated, as they were beyond the scope of this experimental study.

### 2.3 Statistical analysis

Intestinal pressure measurements were obtained from a single group of dogs and cats during two experimental conditions (oral and aboral to the lesion). Each set of measurements was compared with a predefined reference value of 25 mmHg using a two-sided one-sample t-test. Effect sizes were calculated using Cohen’s *d*, and results are presented with 95% confidence intervals. Statistical significance was defined as a *p*-value < 0.05.

## 3 Results

### 3.1 Ex vivo

Thirty-nine intestinal segments obtained from seven fresh cadavers (four dogs and three cats) were evaluated. The mean ILP was 20.7 mmHg (range, 0–198.6 mmHg; 95% CI [5.9–35.5 mmHg]), a value influenced by several high individual measurements. Nevertheless, leakage occurred at pressures below 6 mmHg in 21 of the 39 segments, and 33 of the 39 measurements were below the reported maximal physiological peristaltic pressure of 25 mmHg (Table 1).

**Table 1:** Initial leakage pressure (ILP) values measured in cadaveric jejunal segments from dogs and cats following unsutured full-thickness needle-core biopsy.

Animal	Number of test	ILP (mmHg)
1	4	1.69, 11.27, 13.8, 33.38
2	2	1.05, 1.50
3	6	0.04, 16.02, 4.78, 22.44, 0.58, 19.9
4	5	2.00, 19.02, 11.47, 25.02, 12.68
5	6	23.00, 183.19, 0, 198.63, 30.29, 130.56
6	6	6.17, 1.81, 0, 7.27, 1.29, 1.22
7	10	1.00, 0, 0, 0, 0, 0, 0.58, 25.00, 0, 0.78
Overall	39	Mean = 20.70 mmHg

### 3.2 In vivo

ILP was successfully measured in 12 patients (8 dogs and 4 cats) undergoing enterectomy for unrelated conditions. The animals were presented at a mean age of 5.5 years (0.16–12). The indications for enterectomy were an intestinal mass (*n* = 6), a perforating gastrointestinal foreign body (4), intussusception (1), and intestinal perforation resulting from a gunshot injury (1).

In all animals except one, two measurements were taken. For a single cat, however, only one measurement was taken. In this particular animal, the intestinal lesion was located in the distal jejunum and proximal ileum. Conducting the experiment on the aboral side would have required removing nearly the entire ileum, which could have led to unnecessary complications. Therefore, only one experiment was carried out on the oral/jejunal side for this animal.

**Figure 5:**
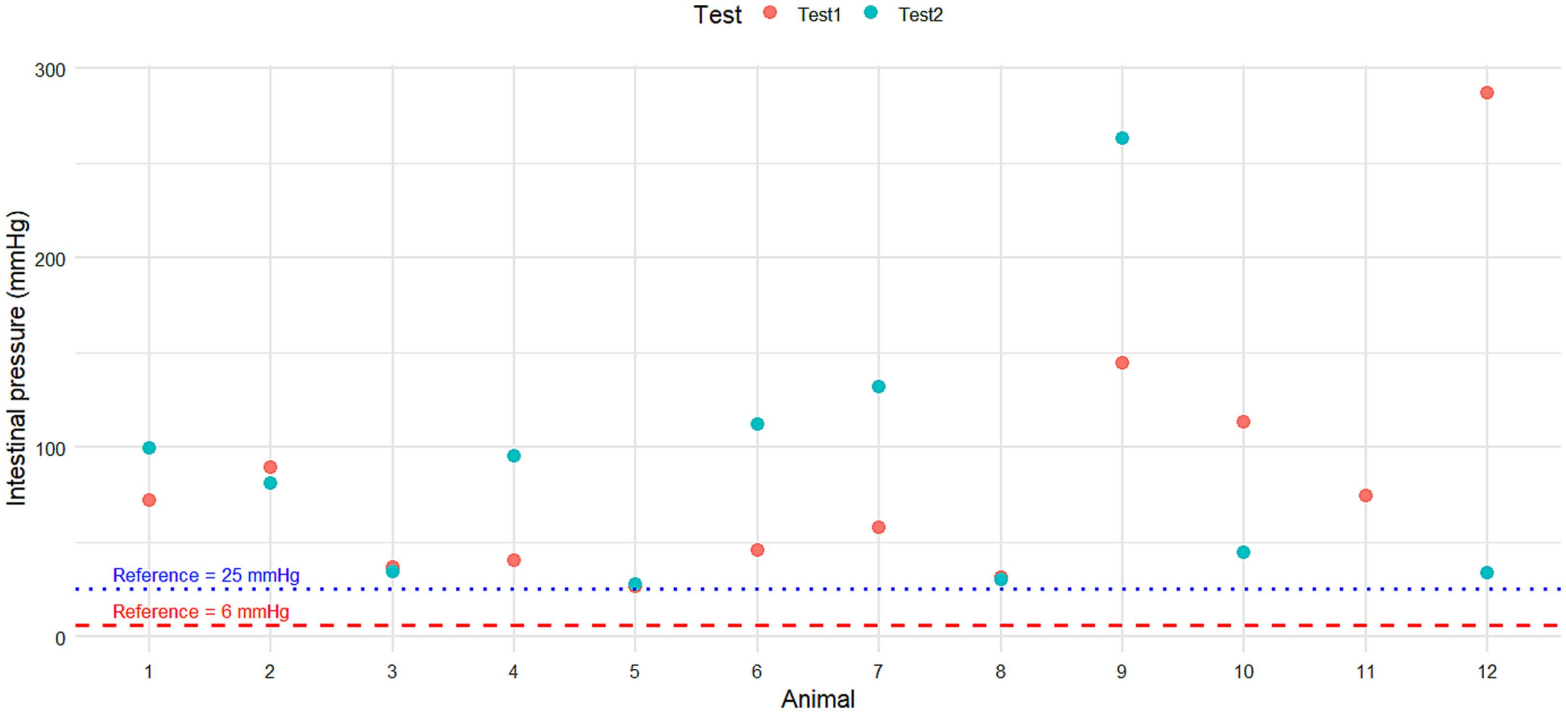
Graph of the intraluminal pressure measurements obtained in 12 animals with two reference values: 6 and 25 mmHg corresponding, respectively, to the mean intestinal pressure and to the maximal intestinal pressure during peristaltic activity.

A total of 23 pressure measurements were obtained. The mean result was 85.66 mmHg (range 26.6–287.7 mmHg) (Table 2). No pressure below 26 mmHg was recorded (Figure 5).

**Table 2:** Initial leakage pressure (ILP) values obtained in live dogs and cats undergoing enterectomy following unsutured full-thickness needle-core intestinal biopsy.

Animal	Number of test	ILP (mmHg)
1	2	72.12, 99.3
2	2	89.2, 81.05
3	2	36.41, 34.52
4	2	40.03, 95.47
5	2	26.63, 27.44
6	2	45.66, 111.85
7	2	57.61, 131.76
8	2	31.00, 29.89
9	2	144.2, 263.33
10	2	113.29, 44.38
11	1	74.29
12	2	287.67, 33.43
Overall	23	Mean = 85.66 mmHg

The one-sample t-test demonstrated that the mean of the combined measurements (mean = 85.66) was significantly different from the reference value of 25 (*t* = 4.18, *p* < 0.001, 95% CI [55.53, 115.78]).

Cadaveric and *in vivo* measurements showed markedly different mean values and variability. Mean ILP values were substantially higher *in vivo* than in cadaveric specimens (85.6 and 20.7 mmHg respectively), with no overlap between the respective 95% confidence intervals, indicating a significant difference between the two models.

## 4 Discussion

The objective of this study was to evaluate the pressure-holding capacity of the intestine following unsutured full-thickness intestinal biopsy performed with an automated needle-core device, by comparing the ILP with reported physiological intraluminal intestinal pressures in dogs and cats (14–18). Our first hypothesis was rejected, as a clear disparity was observed between *ex vivo* and *in vivo* results, emphasizing the importance of evaluating intestinal integrity under physiological conditions. While cadaveric intestinal segments exhibited low resistance to leakage, *in vivo* measurements consistently exceeded maximal physiological thresholds, supporting the second hypothesis that ILP at unsutured needle-core biopsy sites would exceed physiological intraluminal pressures in live animals.

*Ex vivo* testing revealed considerable variability in ILP values, with more than half of the cadaveric segments leaked at pressures well below reported physiological thresholds. These observations likely reflect post-mortem changes in intestinal tissue rather than an inherent inability of biopsy sites to seal. Loss of smooth muscle tone, reduced tissue perfusion, early autolysis, and altered viscoelastic properties of the intestinal wall are known to occur rapidly after death and may significantly compromise mechanical strength (19, 20). Even though specimens were collected within hours after death these physiological alterations are unavoidable. Consequently, *ex vivo* models appear poorly suited for evaluating intestinal leakage pressure and should be interpreted with caution when extrapolating to clinical conditions.

In contrast, *in vivo* testing demonstrated substantially higher ILP values, with no leakage observed below the upper range of physiological peristaltic pressures. The lower bound of the 95% confidence interval exceeds the maximum reported physiological intestinal pressure. No leakage occurred at pressures below physiological levels in any live animal. These results indicate that unsutured biopsy sites created by a 16-gauge automated needle-core device are capable of maintaining short-term intestinal integrity under physiological conditions.

Several mechanisms may explain the high ILP observed *in vivo*. Preservation of intestinal perfusion maintains tissue elasticity and smooth muscle tone, allowing the recoil of the muscularis layer and the partial closure of the biopsy tract. In addition, the biopsy defect was very small (1.3 mm in diameter). Furthermore, the oblique insertion angle of the biopsy needle may have created a valvular tract through the intestinal wall, limiting fluid escape under pressure (7).

Although the immediate pressure resistance observed here is encouraging, it does not establish that suturing can be safely omitted: the greatest risk of dehiscence in gastrointestinal surgery occurs during the lag phase of healing, at approximately 3–5 days after injury, a period that this short-term study did not evaluate. Conversely, several factors could have led to an underestimation of our results. First, the tests were performed on macroscopically healthy tissue located adjacent to a non-viable area requiring surgical resection. At the microscopic level, subclinical alterations may have affected tissue strength.

Furthermore, omentalization has been shown to enhance intestinal integrity following enterotomy (16). This effect was not accounted for in our study, even though it is known to have a beneficial influence *in vivo*. Finally, in our experiments, the intestines were tested immediately after the biopsy procedure. In clinical conditions, however, intestinal distension would not occur right away, and the formation of a platelet clot at the biopsy site could substantially increase the resistance to leakage, leading to higher pressure values than those measured in our study.

The clinical implications of these findings are potentially significant. Full-thickness intestinal biopsies require laparotomy or laparoscopic-assisted techniques, which may be invasive for diagnostic procedures alone (5–8). Those procedures are associated with surgical morbidity and mortality (9–11). Demonstrating that small (1.3-mm) needle-core biopsy sites can withstand intraluminal pressures above physiological levels immediately after biopsy may support the development of less invasive diagnostic approaches. Techniques such as laparoscopic, laparoscopic-assisted, percutaneous, or ultrasound-guided intestinal biopsies could become safer and more practical if routine closure of biopsy sites proves unnecessary. Reduced operative time, decreased tissue manipulation, and minimized surgical trauma could translate into shorter anesthesia duration and improved postoperative recovery.

One of the main limitations of this study is that the intestines examined were macroscopically normal tissue. Diseased intestines, affected by inflammation or neoplasia, may exhibit altered wall strength and healing capacity. As such, the sealing ability observed in this study may not be directly applicable to all pathological conditions encountered in clinical practice. A clinical investigation evaluating the tolerability and safety of this technique in pathological intestines would therefore be valuable.

Additionally, the controlled and progressive nature of pressure infusion may not perfectly replicate dynamic physiological conditions, where pressure fluctuations are intermittent rather than continuous. Furthermore, a feasibility study on fully laparoscopic intestinal biopsies is warranted. Such a study should determine whether this approach enables consistent and straightforward sampling, and whether an appropriate biopsy angle can be achieved laparoscopically, without increasing the risk of injury to the mesenteric border.

In conclusion, this study provides experimental evidence that unsutured full-thickness intestinal biopsies performed with an automated needle-core biopsy device can withstand intraluminal pressures exceeding physiological levels *in vivo*, indicating a low immediate risk of digestive leakage even without surgical closure of the biopsy site. However, long-term healing was not assessed in the present study, so whether closure of these sites can be safely omitted in clinical practice remains uncertain. Further investigations are warranted, particularly to evaluate healing during the critical 3–5 day postoperative period and to determine the safety of this approach in diseased intestine.

## Data Availability

The original contributions presented in the study are included in the article/supplementary material, further inquiries can be directed to the corresponding author.
